# Prevalence and associated factors of hypertension among adults in Gondar, Northwest Ethiopia: a community based cross-sectional study

**DOI:** 10.1186/1471-2261-12-113

**Published:** 2012-11-28

**Authors:** Akilew Awoke, Tadesse Awoke, Shitaye Alemu, Berihun Megabiaw

**Affiliations:** 1 Department of Epidemiology and Biostatistics, Institute of Public Health, College of Medicine and Health Sciences, University of Gondar, Gondar, Ethiopia; 2 Department of Internal Medicine, School of Medicine, College of Medicine and Health Sciences, University of Gondar, Gondar, Ethiopia

**Keywords:** Hypertension, Prevalence, Blood Pressure, Ethiopia

## Abstract

**Background:**

Hypertension is a growing public health problem in many developing countries including Ethiopia. However, its prevention and control has not yet received due attention. This study aimed to determine the prevalence and associated factors of hypertension among adults in Gondar city, North-West Ethiopia.

**Methods:**

A community based cross-sectional study was conducted in April 2012 in Gondar city. Participants aged 35 years and older were recruited using multi-stage random sampling technique. Data were collected by face-to-face interview technique after verbal informed consent. Additionally, weight, height and Blood Pressure (BP) of participants were measured following standard procedures. Hypertension was defined as having Systolic BP ≥140 mmHG or Diastolic BP≥ 90mmHG or reported use of regular anti-hypertensive medications prescribed by professionals for raised BP. Data were collected by clinical nurses and then entered into a computer using Epi Info version 3.5.3 and exported to SPSS version 20 for analysis. Multiple logistic regressions were fitted and Odds ratios with 95% confidence intervals were calculated to identify associated factors.

**Results:**

A total of 679 participants were included in this study. About one in –five participants (21.0%) were aged 65 years or older. Obesity among all participants was 5.6%. Hundred ninety two (28.3%) were hypertensive of whom more than a third (37.0%) did not know they had hypertension. Family history of hypertension (AOR = 2.71, 95%CI; 1.37-5.36), obesity (AOR = 5.50, 95%CI; 2.07-14.62), self reported diabetes (AOR = 4.15, 95%CI; 1.77-9.72), age ≥ 55 years (AOR=3.33, 95%CI; 1.88-5.90) and not continuously walking for 10 minutes per day (AOR = 2.86, 95%CI; 1.15-7.12) were factors associated with hypertension.

**Conclusion:**

There was a high prevalence of hypertension probably indicating a hidden epidemic in this community. Age ≥ 55 years, obesity, family history of hypertension, physical inactivity and self reported diabetes were associated with hypertension. Hence, we recommend the design and implementation of community based screening programs.

## Background

Raised blood pressure is estimated to cause 7.5 million (12.8% of all causes of death) deaths per year. Hypertension (HTN) doubles the risk of cardiovascular diseases such as coronary heart diseases (CHD), congestive heart failure (CHF), stroke, renal failure, and peripheral arterial diseases. Prevalence of hypertension varies among nations and sub-populations within a nation though generally lower among high-income populations [1-3]. Mass migration from rural to urban areas and lifestyle changes associated with “civilization” may explain the apparently rising prevalence of hypertension in urban populations. Hypertension rapidly increased from 3% in rural areas to 30% in some urban settings. In some populations, hypertension prevalence rates were higher in women than in men while the opposite was true in others. Both lower-income as well as -higher income groups are at increased risk of developing hypertension [4].

The prevention and control of hypertension has not received due attention by many developing countries. Hypertension is one of the most modifiable risk factors for cardiovascular diseases. However, awareness about treatment and control of hypertension is extremely low among developing nations including Ethiopia. In these countries health care resources are overwhelmed by other priorities including HIV/AIDS, tuberculosis, and malaria [5]. Little is known about the magnitude and determinants of hypertension in Ethiopia particularly in the study area. However, recent evidences indicate that hypertension and raised blood pressure are increasing partly because of the increase in risk factors including smoking, obesity, and harmful use of alcohol and lack of exercise [5].

Thus, this study aimed to identify the prevalence and associated factors of hypertension among adults in Gondar city.

## Methods

A community based cross-sectional study was conducted in April 2012 in Gondar city. Gondar city is located 750 kilometers North-West from Addis Ababa, the Ethiopian capital. The city has 21 *kebeles* (the smallest administrative units in Ethiopia) and is among the ancient and densely populated cities in Ethiopia having 206,987 people according to 2007 Ethiopian Central Statistical Agency (CSA) office report [6].

The study included 679 permanent residents (who lived in the area at least for 6 months) aged 35 years or older selected using a multistage random sampling technique. In the first stage, three out of 12 *kebeles* (25% of the total area) were selected by simple random sampling technique. In the second stage, a total of 696 households were selected using a systematic random sampling method. In this process samples were proportionally allocated to each selected *kebeles*. Total number of households was obtained from the respective administrative areas and used to calculate the sampling fraction. Only one eligible individual was interviewed from the selected household. Occasionally, when two or more individuals were eligible in a household, only one was selected by lottery method. Three pregnant ladies were excluded from the study.

The interview questionnaire was structured into three logical sections (socio demography characteristics, behavioral and medical related questions, and measurements). The WHO STEPS instrument [7] was adopted to collect data on selected behavioral and lifestyle characteristics and measurements of weight and height.

### Terms and definitions

Hypertension was defined as a sustained high blood pressure (SBP≥140 or DBP ≥90mmHg) [3] or reported regular use of anti-hypertensive medication(s). Body Mass Index (BMI) was calculated as weight in kilograms divided by height in square meters and interpreted as underweight (BMI<18.5), normal (18.5 - 24.9), overweight (25.0 - 29.9) and obese (≥ 30.0).

### Data collection

Data were collected using a combination of a structured questionnaire and measurements of weight, height and Blood Pressure (BP). Data collectors were six clinical nurses supervised by investigators. Training and practical demonstrations on interview techniques and measurement procedures were given to data collectors for two consecutive days.

The questionnaire was pretested on 5% of the study participants found outside of the study area and modifications were made on the basis of the findings. After completing the interview, the participant’s height and weight were measured and recorded by interviewers. Weight measuring scales were checked and adjusted at zero level between each measurement and height was measured following the standard steps. Blood pressure was measured twice in a sitting position using standard mercury sphygmomanometer BP cuff with the appropriate cuff size that covers two-thirds of the upper arm after the participant rested for at least five minutes and no smoking or caffeine 30 minutes before measurement. The second measurement was taken five-to-ten minutes after the first measurement. Finally the average of the two BP measurements was calculated to determine the BP status of the participant.

Statistical analyses were done by bivariate and multivariate methods. Chi- squared tests were used when comparing groups. All factors with a p-value <0.2 in the bivariate logistic regression analysis were further entered into the multivariate model to control confounding effects.

The Hosmer -Lemeshow goodness-of- fit statistic was used to assess whether the necessary assumptions for the application of multiple logistic regression are fulfilled. Odds ratios (OR) with 95% confidence intervals (CI) were calculated. Statistical significance was accepted at the 5% level (p<0.05). Ethical clearance was obtained from the University of Gondar, Institute of Public Health. Permission letter was obtained from Gondar City administration office. Respondents were fully informed about the purpose of the study and gave verbal consent. Confidentiality of the information was assured from all the data collectors and principal investigators side. Participants having hypertension by our measurement were referred to nearby health facilities for further diagnosis and treatment.

## Results

A total of 679 adults (97.6% response rate) were included in this study. More than half (52.4%) were females. The mean age was 51.5 +/−14.4 years. Majority (81.8%) were Orthodox Christians and Amhara ethnics (73.9%). Nearly half (52.3%) were married and about one third (35.3%) were housewives (Table 1).

**Table 1:** Socio-demographic characteristics of the study participants in Gondar city, North West Ethiopia, May 2012(n= 679)

**Characteristics**	**Frequency**	**Percent**
Sex		
Male	323	47.6
Female	356	52.4
Age		
35-44	263	38.7
45-54	162	23.9
55-64	109	16.1
>=65	145	21.4
Marital status		
Single	73	10.8
Married	355	52.3
Divorced	106	15.6
Widowed	145	21.4
Education level		
No formal education	245	16.1
Primary level	157	23.1
Secondary level	172	25.3
Tertiary level	105	15.5
Occupation		
Government employed	107	15.8
Merchant	127	18.7
Daily laborer	98	14.4
House wife	240	35.3
Retired	70	10.3
Others*	37	5.4
Religion		
Orthodox	556	81.9
Muslim	112	16.5
Protestant	7	1.0
Others**	4	0.6
Ethnicity		
Amhara	569	83.8
Tgrie	62	9.1
Kimant	40	5.9
Others	8	1.2
Monthly income		
≤ 750	173	25.5
751-1300	165	24.3
1301-2000	187	27.5
>2000	154	22.7

*= Drivers, farmers **= Catholic, no religious affiliation.

Thirty two participants (4.7%) declared that they were smoking cigarettes previously. Nineteen participants (2.8%) were current smokers of whom seven (36.8%) were smoking at least half a pack (10 or more cigarettes) every day. Concerning their alcohol use, 251(37.0%) were current users.

One from every seven participants (16.9%) was involved in vigorous activities such as carrying or lifting heavy loads, and construction works. Nevertheless, only 24(20.9%) do so on a daily basis. Most participants (95%) use to walk for at least 10 minutes continuously every day. More than three quarters (76.9%) of participants reported using vehicles as main mode of transportation to their work places (Table 2).

**Table 2:** Hypertension prevalence across behavioral and dietary related characteristics of respondents in Gondar city (n= 679) May 2012

**Characteristics**	**n**	**Percent**	**Hypertension (%)**
**Smoking**			
Never	628	92.5	28.8
Current	19	2.8	15.8
Previously	32	4.7	25.0
**Alcohol use**			
Never	360	53	28.9
Current	251	37	24.3
Previous	68	16	39.7
**Commonly used oil/fat**			
Saturated oil	460	67.7	27.2
Sesame/nug oil	212	31.2	31.1
Others	7	1.0	14.3
**Fruit consumption/week**			
None	340	50.1	28.8
1-3days	317	46.7	28.1
4-7days	22	3.2	22.7
**Vegetable use/week**			
None	134	19.7	34.3
1-3days	459	67.6	24.4
4-7days	86	12.7	39.5
**Excessive salt**			
Yes	143	21.1	33.6
No	536	78.9	26.9
**Vigorous work/week**			
None	564	83.1	29.6
1-3	33	4.9	18.2
4-7	82	12.0	23.2
**Walking status**			
None	34	5	55.9
Daily	479	70.5	30.5
<daily/wk	166	24	25.7
**Transportation**			
Use vehicle	522	76.9	29.9
On foot	157	23.1	27.8
**Diabetes status**			
Yes	47	6.9	66.0
No	632	93.1	25.5
**Family History of hypertension**			
Yes	81	11.9	44.4
No	598	88.1	26.1

### Dietary habits of respondents

About two third (67.7%) of respondents reported that they usually use vegetable oil for meal preparation while a similar proportion (67.6%) reported eating vegetables at least 1–3 days in a week. Half of the respondents (50.1%) do not eat fruits at all in any days of a week. Hundred and forty three (21.1%) respondents have reported excessive use of salt than other family members (Table 2).

### BMI of respondents

The mean BMI of respondents was 23.35 (±4.02 SD) kg/m^2^. One quarter (25.3%) of participants was overweight while 5.6% were obese.

### Prevalence of hypertension

The mean systolic and diastolic BP results were 120.2mmHg (±19.6 SD) and 72.4 mmHg (±10.5 SD). The overall prevalence of hypertension was 28.3% (95% CI: 24.9-31.7), slightly lower in men (26.0%) than women (30.3%) though the difference was not statistically significant (X^2^=1.57, P= 0.211). Among all hypertensive people identified, 71(37.0%) did not know they had hypertension (newly screened). Of the 121(63.0%) hypertensive people who reported using anti-hypertensive medications during data collection period, 42.15% had normal BP on measurement.

### Factors associated with hypertension

Among the non-modifiable factors, age and family history of hypertension were associated with hypertension. The likelihood of hypertension increased with advancing age. Among subjects aged 55 years and above, the AOR was 3.33 [95%CI: 1.88-5.90] as compared to those 35–44 years old. Participants with family history of hypertension were almost three times (AOR= 2.71 &95%CI, 1.37-5.36) at increased risk of hypertension compared to their counterparts. If participants had self-reported diabetes, then they were about four times (AOR=4.15 & 95%CI 1.77-9.72) more likely to be hypertensive also. Participants who did not walk at least for 10 minutes continuously on daily basis were about three times (AOR=2.86 & 95%CI 1.15 -7.12) highly likely to be hypertensive. Being obese (AOR=5.50 &95%CI: 2.07-14.62) was significantly associated with hypertension compared to having normal BMI. In this particular study, risky behaviors including smoking and alcohol use were not significantly associated with hypertension (Table 3).

**Table 3:** Bivariate and multivariate logistic regression analysis of factors associated with hypertension among study participants in Gondar city (n=679), May 2012

**Variables**	**Hypertension**		**Crude OR**	**Adjusted OR**
	**Yes**	**No**	**(95% CI)**	**(95% CI)**
**Age**				
35-44	41	222	1.00	1.00
45-54	39	123	1.72 (1.05-2.80)*	1.41 (0.74-2.69)
≥ 55	112	142	4.27 (2.82-6.47)*	3.33 (1.88-5. 90)*
**Education level**				
No formal education	85	160	1.00	1.00
Primary level	43	114	0.71 (0.46-1.10)	0.61 (0.31-1.22)
Secondary	41	131	0.59 (0.38-0.91)*	0.89 (0.44-1.81)
Tertiary	23	82	0.53 (0.31-0.89)*	0.85 (0.34-2.11)
**Marital status**				
Single	21	52	1.00	1.00
Married	83	272	0.76 (.43-1.33)	0.53 (0.23-1.24)
Divorced	23	83	0.69 (.35-1.36)	0.52 (0.20-1.38)
Widowed	65	80	2.01 (1.10-3.68)*	0.77 (0.31-1.89)
**Occupation**				
Government employed	22	85	1.00	1.00
Merchant	28	99	1.09 (0.58-2.05)	1.02 (0.37-2.85)
Daily laborer	17	81	0.81 (0.40-1.64)	1.18 (0.37-3.76)
House wife	87	153	2.20 (1.28-3.76)*	1.33 (0.52-3.44)
Retired	28	42	2.58 (1.32-5.03)*	0.92 (0.29-2.90)
**Others	10	27	1.43 (0.60-3.39)	1.05 (0.21-5.13)
**BMI**				
Normal	101	298	1.00	1.00
Underweight	10	60	0.49 (0.24-0.99)*	0.50 (0.22- 1.16)
Overweight	58	114	1.50 (1.02-2.21)*	1.57 (0.93- 2.64)
Obese	23	15	4.52 (2. 27–9.01)*	5.50 (2.07-14.62)*
**Self reported DM**				
Yes	31	16	5.67 (3.02-10.64)*	4.15 (1.77-9.72)*
No	161	471	1.00	1.00
**Family history of hypertension**				
Yes	36	45	2.27 (1.41-3.64)*	2.71 (1.37-5.36)*
No	156	442	1.00	1.00
**Vegetable use/week**				
None	46	88	0.80 (0.46-1.40)	0.70 (0.30 -1.65)
1-3days	112	347	0.49 (0.31-0.80)*	0.55 (0.26 -1.18)
4-7days	34	52	1.00	1.00
**Walking status for 10 minutes**				
Yes	173	472	1.00	1.00
No	19	15	3.46 (1.72-6.96)	2.86 (1.15 -7.12)

*= P-value < 0.05 **=drivers &farmers, (backward logistic regression method was employed).

## Discussion

Hypertension is among the leading causes of death globally. It increases risks of stroke, heart diseases, kidney failure and other diseases [1,3]. Slightly more than one in every four adults aged 35 years or older had hypertension in Gondar city (28.3%). This prevalence is high and may be considered as a major public health problem in this community. As a result complications of hypertension such as disabilities are more likely to occur in this community particularly among those not aware of being hypertensive [8].

This result was comparable with a community-based study conducted in Addis Ababa, Ethiopia which reported a 31.5% and 28.9% prevalence of hypertension among males and females, respectively [9]. It is also comparable with a 29.3% prevalence report from the United States [10]. This may be due to the fact that the prevalence of hypertension in this country is getting due attention only recently and may still be raising while that of the USA is declining with treatment and life style modifications.

Prevalence of hypertension in this study is considerably higher as compared to previous reports from southern Ethiopia (10.1%), Vietnam (14.1%) and Jordan (23.9%) [11-13]. This discrepancy may be explained in two ways; First, the age difference in the study populations (>=35 years in our case while in other studies age of participants ranges between 18–65 years). Secondly, this study was conducted only in an urban setting.

This prevalence was lower than other similarly community based studies conducted in Uganda, Mozambique, Eastern Nigeria and Northern India in which the prevalence of hypertension ranged from 30.5 to 44.5%) [14-17]. This difference may be attributable to higher prevalence of obesity (Nigeria, 13.3%) on top of this, racial and genetic difference which could probably affect blood pressure.

As many studies agreed, there is a positive association between age and hypertension [12,13,15,18] in which the risk of hypertension increases with age. This is mainly due to arterial stiffness as one gets older. Hypertension was 9% higher among those aged 45–54 years compared to those 35–44 years old, while it increased by 20% in those 55 years or older compared to those 45-54 years of age category. In the current study, obese people had a 5.5 times higher risk of hypertension compared to those with normal BMI. Obesity was more prevalent among females but its effect on hypertension was more pronounced among males (76.3% and 23.7% of obese males and females were hypertensive respectively). This made hypertension more or less similar across both sexes. This finding was in line with previous reports from Ethiopia, Eretria, Uganda and India [9,17-20]. Similar to other previous studies reported so far [11,12,21], family history of hypertension was significantly associated with being hypertensive in this study.

People who reported having diabetes were about 4 times more likely to have hypertension as well. In this study, 66% of those who reported being diabetic had hypertension. This might be due to the fact that both diseases share common risk factors and/or due to the fact that the two conditions (diabetes and hypertension) may cause each other [2,3]. This finding is in agreement with several other studies [12,19,20]. This study also revealed that walking for at least ten minutes continuously everyday was negatively associated with hypertension. This study further strengthens the previous reports in this country [9,11].

Unlike other studies done so far [11,16,17,22], cigarette smoking, harmful use of alcohol and excessive use of salt were not significantly associated with hypertension in this study. This may be due to the low prevalence of these factors in the community studied.

This study has potential limitations. Firstly, being a cross-sectional one it has inherent limitation; hypertension might have preceded some of the explanatory variables. Second, this study is limited to behavioral and physical measurements, and did not include biochemical measurements such as a 24 hours urine sodium concentration, serum glucose level, etc. Thirdly, it was only limited to adults aged 35 or older which made comparisons with other studies difficult.

## Conclusion

There was high prevalence of hypertension among adults in Gondar and may show a hidden epidemic in this population. A significant proportion (37%) of participants were unaware of having the condition (screened newly for the first time) before they were identified by the current study.

Family history of hypertension, self reported diabetes, obesity, physical inactivity and age were associated factors with hypertension. Hence, we recommend the design and implementation of community based screening programs for hypertension in this community.

## Competing interests

The authors declare that they have no conflict of interests.

## Authors’ contributions

AA wrote the proposal, participated in data collection, analyzed the data and drafted the paper. BM and TA approved the proposal with some revisions, participated in data collection, analysis and manuscript writing. SA participated in data collection and manuscript editing. All authors read and approved the final manuscript.

## Pre-publication history

The pre-publication history for this paper can be accessed here:

http://www.biomedcentral.com/1471-2261/12/113/prepub
